# Periosteal distraction as a new surgical technique for the treatment of senile diabetic foot: A retrospective case analysis

**DOI:** 10.1097/MD.0000000000041183

**Published:** 2025-01-03

**Authors:** Rui Yang, Yue Zhou, Yunxiang Hu, Sanmao Liu

**Affiliations:** a Department of Orthopedics, The First Affiliated Hospital of Guangxi Medical University, Nanning, Guangxi Zhuang Autonomous Region, P.R. China; b School of Graduates, Dalian Medical University, Dalian City, Liaoning Province, China; c Department of Orthopedics, The Second Affiliated Hospital, Dalian Medical University, Dalian, Liaoning, P.R. China; d Department of Orthopedics, Central Hospital of Dalian University of Technology, Dalian City, Liaoning Province, China.

**Keywords:** diabetic foot, periosteal distraction technique, periosteum, tibia.

## Abstract

This study investigates the clinical effect and safety of periosteal distraction in the treatment of senile diabetic foot. The clinical data of 45 patients with diabetic foot treated with periosteal distraction in the Central Hospital of Dalian University of Technology from January 2020 to May 2024 were retrospectively analyzed. Finally, 42 patients were followed up, and 3 patients were lost to follow-up, including 29 males and 13 females, aged (71.17 ± 6.43), (62–84) years respectively. The Wagner grade of the ulcer surface of the affected foot was grade 2 in 25 cases, grade 3 in 13 cases, and grade 4 in 4 cases; the ulcer sites were toes in 18 cases, soles in 14 cases, dorsum of the foot in 8 cases, heels in 1 case, and ankles in 1 case. The toe oxygen saturation, ankle-brachial index (ABI), skin temperature and visual analogue score (VAS) were recorded before operation and at 1 day, 7 days, 14 days, 1 month, 2 months, and 3 months after operation. The therapeutic effect was observed and evaluated in combination with Michigan neurological sign score and lower limb computed tomography angiography. The wound ulcer healing rate, amputation rate and ulcer recurrence were also counted. The toe oxygen saturation, ABI, skin temperature, VAS score and Michigan neurological sign score of all patients were significantly improved after operation compared with those before operation, and the differences were statistically significant (*P* < .05); After a 3-month post-surgery period, 37 patients were observed to have microcirculation formation in the affected limb, as well as increased and thickened lower extremity arterioles in comparison to pre-surgery conditions, forming an interwoven network. During the follow-up period, 21 patients exhibited healed foot ulcers at 2 months post-surgery, while by the end of the follow-up period, 38 patients had healed foot ulcers, 5 patients had experienced a significant reduction in wound ulcer area, and the overall ulcer healing rate was 90%. Notably, no instances of amputation or ulcer recurrence were observed during treatment. Periosteal distraction is a new surgical method for the treatment of senile diabetic foot ulcer, which can obtain satisfactory short-term efficacy and is worthy of clinical promotion.

## 1. Introduction

**Figure 1.**
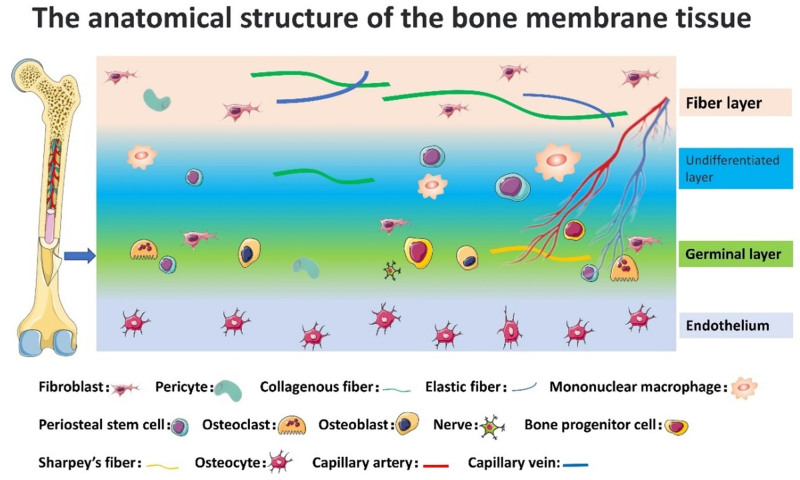
The periosteum can be divided into the inner periosteum and outer periosteum, which connects with the cortical bone and shows a large number of osteocytes; the periosteum usually refers to the outer periosteum and is divided into 3 layers, that is, the fibrous layer, the undifferentiated layer and the germinal layer from outside to inside, of which the fibrous layer is mainly thick collagen fiber bundles; the main cellular component of the undifferentiated layer is undifferentiated cells, which can provide progenitor cells for the fibrous layer and the germinal layer; the blood vessels and cells in the germinal layer are abundant, and their cellular components are osteoprogenitor cells, osteoblasts, osteoclasts and vascular endothelial cells, so this layer has osteogenic potential.

Diabetic foot is a significant global medical, social and economic problem. It is the most common primary endpoint of diabetic complications and 1 of the major risk factors for amputation. Diabetic neuropathy and peripheral vascular disease are the main causes of foot ulcers and may act alone or in combination with other factors (e.g., microvascular disease, biomechanical abnormalities, limited joint mobility, and increased susceptibility to infection).^[1]^ Studies have shown that diabetic patients have a 15% to 25% chance of developing diabetic foot ulcers (DFUs) during their lifetime, with a recurrence rate of 50% to 70% within the following 5 years.^[2]^ Hence, timely intervention and management of diabetic foot is imperative, and it constitutes a significant concern for diabetologists, internists, and surgeons.^[3]^ Currently, the clinical management of diabetic foot encompasses medical management, surgical management, and percutaneous transluminal angioplasty for stenosed and occluded lower extremity arteries. Medical management is often used in the early stages of ulcer formation, focusing on blood glucose and blood pressure control, infection prevention, plantar offloading, advanced dressings, oxygen therapy, negative pressure treatment, and newer innovations like stem cell, gene, and nanotherapy.^[4–18]^ Surgical options include arterial reconstruction, sympathetic nerve resection, and amputation.^[19–23]^ However, these methods have limitations, and diabetic foot prevention and treatment remain active areas of research. We introduce a novel surgical option for DFUs: the membrane distraction technique. Developed by Chinese scholar Zeng Naxin,^[24]^ this technique leverages the tensile force from tibial transverse bone transfer to promote angiogenesis.^[25,26]^ The periosteum, a connective tissue membrane covering bones (excluding joints), contains rich microvessels and osteogenic cells. It consists of 3 layers – fibrous, undifferentiated, and germinal – without clear boundaries^[27]^ (Fig. 1). The membrane distraction technique applies mechanical traction to periosteal cells, converting mechanical signals into biochemical responses. This process enhances gene expression and cellular function, leading to new blood vessel formation around the traction site, thus improving blood supply to treat DFUs. While this technique has been reported as effective in domestic journals, it is underrepresented in English-language literature. Our hand and foot surgery department has been a pioneer in this method, accumulating significant clinical experience. We aim to compile and analyze recent cases to share our insights on the membrane distraction technique for treating DFUs with the global medical community.

## 2. Materials and methods

### 2.1. General data

A retrospective study was conducted on the clinical data of 42 patients with diabetic foot treated with periosteal distraction technique in the Affiliated Central Hospital of Dalian University of Technology from January 2020 to May 2024. Among them, there were 29 males and 13 females, aged (71.17 ± 6.43) (62–84) years. According to Wagner classification,^[28]^ the ulcer surface of all affected feet included 25 cases of grade 2, 13 cases of grade 3, and 4 cases of grade 4; According to the classification of foot ischemia severity, diabetic foot can be categorized into ischemic, neuropathic, and neuroischemic (mixed) types.^[29]^ In this study, there were 36 cases of ischemic type and 6 cases of mixed type, with no cases of purely neuropathic type included. The ulcer sites were toes in 18 cases, soles in 14 cases, dorsum of the foot in 8 cases, and heels and ankles in 1 case each. Informed consent was obtained from all patients; this study was approved by the ethical committee of Dalian Municipal Central Hospital (number: YN2023-060-01).

### 2.2. Inclusion criteria and exclusion criteria

Inclusion criteria: According to the diagnostic criteria of type 2 diabetes,^[30]^ patients were diagnosed with diabetic foot; patients were aged ≥ 60 years; Wagner grade 2 or higher, that is, accompanied by bone tissue lesions or abscesses and even gangrene; Cases were classified as ischemic type or neuroischemic type based on the degree of foot ischemia; computed tomography angiography (CTA) revealed patency of at least 1 of the 3 major vessels below the knee of the affected limb (anterior tibial artery, posterior tibial artery, and peroneal artery) to the ankle joint level. Ulcers have not healed or recurred for at least 1 month; patients have not undergone other treatments such as vascular intervention; agree to participate in this study.

Exclusion criteria: foot ulcers caused by non-diabetes; cases were classified as Wagner grade 1 or as purely neuropathic type based on the degree of ischemia; coupled with significant comorbidities that preclude surgical intervention.; incomplete clinical data; patients lost to follow-up. Patients who experience mental illness or severe disruptions of consciousness and exhibit an inability to comply with treatment.

### 2.3. Preoperative preparation

All patients were strictly controlled under the guidance of surgeons, preoperative blood glucose levels should meet fasting blood glucose levels < 7.8 mmol/L, postprandial blood glucose < 10 mmol/L^[31]^; Normal saline was utilized for wound cleansing and necrotic tissue removal, followed by debridement for patients necessitating the procedure. A bacterial culture was obtained from the wound surface of the affected limb, and antibiotics with sensitivity to the culture results were chosen.

### 2.4. Surgical procedure

**Figure 2.**
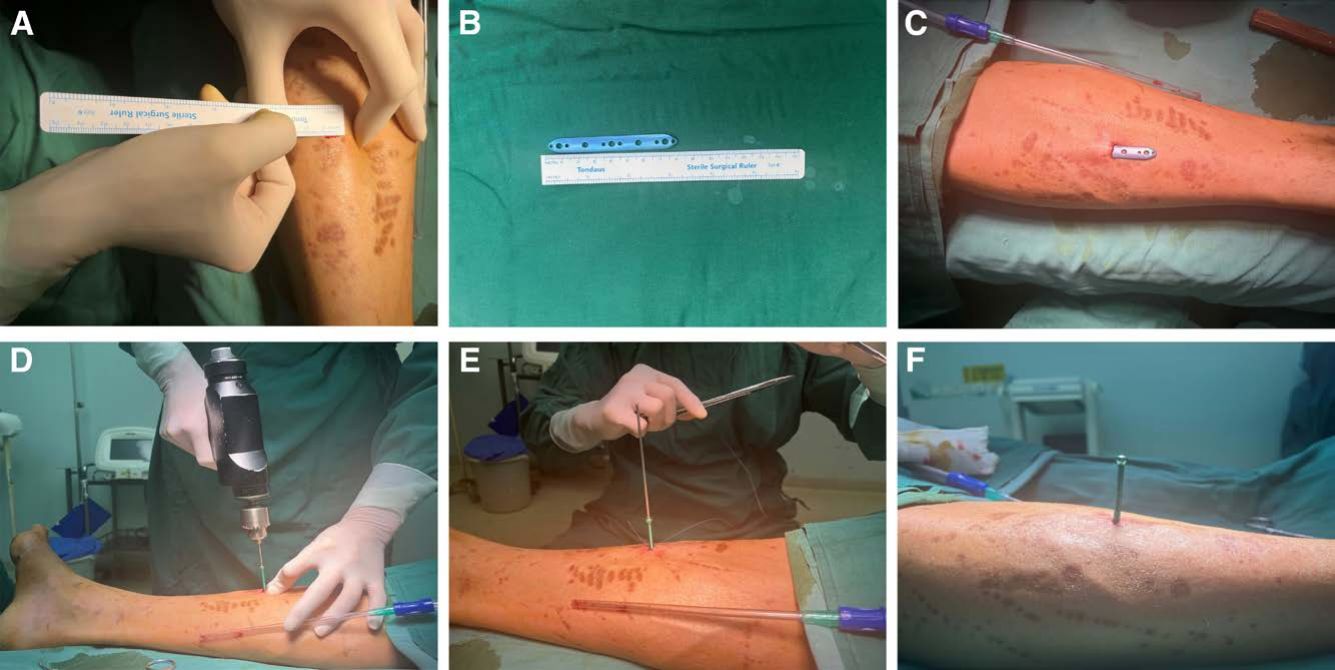
Implantation of the periosteal distraction locking plate system. (A) transverse skin incision for periosteal distraction, about 1 cm in length; (B) length of periosteal distraction plate, about 8 cm; (C) implantation of periosteal distraction plate between periosteum and cortical bone; (D) 1 fixed periosteal distraction plate with cannulated screws and Kirschner wires; (E) suture soft tissue layer by layer; (F) cannulated screws for distraction compression.

After the combined block anesthesia was significantly effective, the patient was placed in the supine position, the affected limb was routinely disinfected and draped, and the operation was started. A transverse incision (which can be a longitudinal incision) about 1 cm in length was made in the medial aspect of the middle leg of the affected limb, and the subcutaneous skin was incised layer by layer and hemostasis was performed along the way with an electrocautery, and after the periosteum was incised, the periosteum was appropriately dissected with a special periosteal elevator extending the medial aspect of the middle tibia. After confirming that the gap is reliable, a distraction periosteal distraction locking plate (periosteal distraction plate) is placed, and 1 cannulated screw and Kirschner wire were used for reliable fixation. C-arm fluoroscopy showed ideal length of implant position. Subsequent to the biting off of the vegetation and Kirschner wire, the surgical area was irrigated and a full thickness suture was applied using 3 to 0 absorbable suture. A sterile dressing was subsequently administered. The wound surface of the affected limb was cleansed, and curettage was executed to eliminate necrotic fascial tissue and expose blood exudation. The area was then enveloped with paraffin gauze and a sterile dressing compression bandage was implemented (Fig. 2).

### 2.5. Postoperative treatments

**Figure 3.**
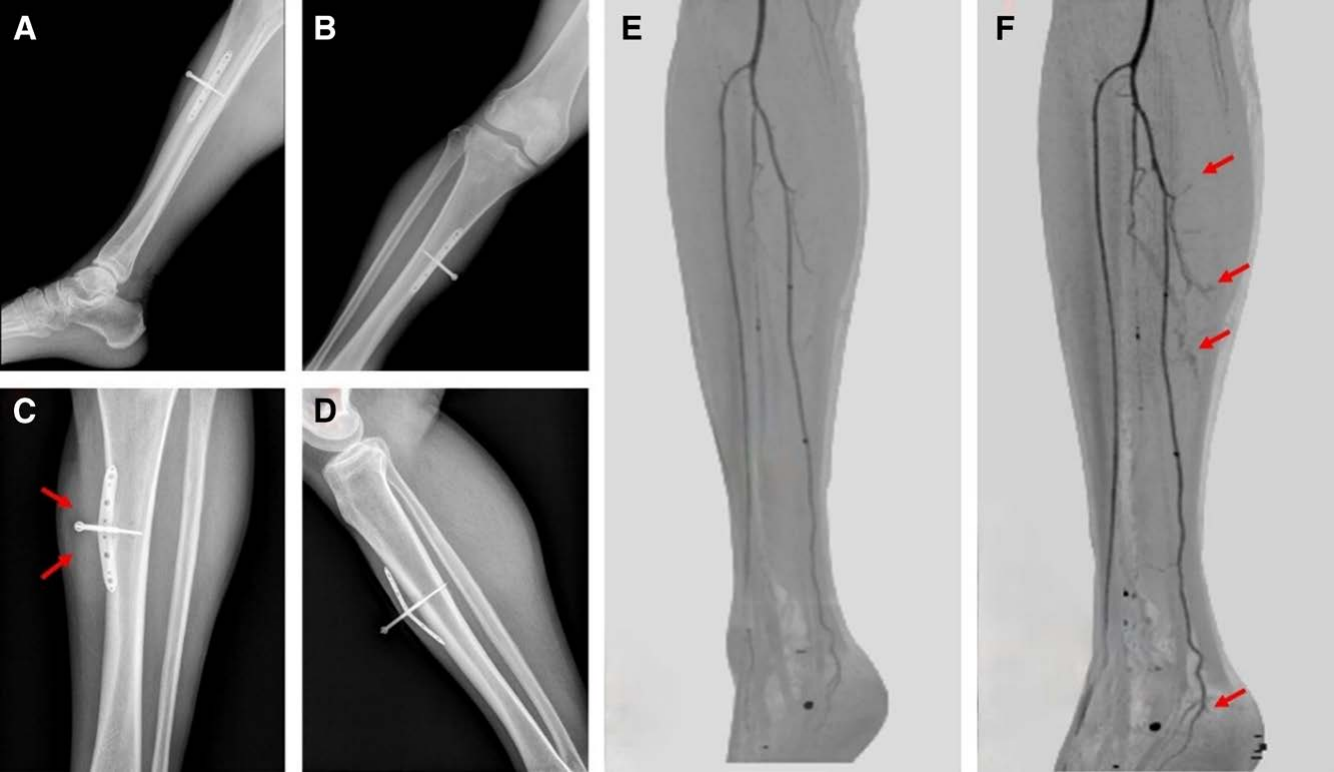
Periosteal distraction process and CTA vascular visualization of the affected limb before and after surgery. (A and B) refers to the DR condition of the affected limb on the first day after periosteal distraction, periosteal distraction plate is located in the middle and upper tibia, abutting the bone surface; (C and D) refers to the DR condition of the affected limb on the 14th day after periosteal distraction, periosteal distraction plate has bulged the bone surface with the continuous rotation of cannulated screws, so that the periosteum is continuously distracted, (C) (red arrow) showed soft tissue thickening, and inflammatory shadows; (E and F) refers to the CTA vascular development contrast of the affected limb before surgery (E) and 3 mo after surgery (F), in (F) (red arrow) showed that the vascular development of the affected limb 3 months after surgery has significantly increased and thickened compared with that before surgery, and formed an interwoven network, meanwhile, the closer to the periosteal distraction site, the denser the neovascular network, also a few neovascular shadows were also observed in the heel (red arrow). CTA = computed tomography angiography.

Antibiotics were selected for treatment according to the results of preoperative and drug sensitivity tests. The dressing was changed on time and the screw orifice was cleaned, and the internal fixation status was observed by X-ray 2 weeks after surgery. The technique of periosteal distraction was initiated on the second day of treatment. The screws were rotated through a screw cap, resulting in a daily outward distraction of 1 mm. This process was repeated 2 times per day, with intervals of 12 hours, for a total of 14 days. Following this period, a 3-day rest period was observed, and the distraction device was subsequently removed. The wound was promptly dressing changed in order to maintain cleanliness. Patients were monitored perioperatively and insulin was used to control blood glucose stability (fasting blood glucose < 8 mmol/L). Michigan score at 3 months after surgery, and CTA of the affected limb were used to assess the postoperative effect (Fig. 3).

### 2.6. Three-dimensional images of CTA

Three-dimensional images of CTA were obtained using a Siemens 128-slice multi-slice spiral computed tomography(MSCT) scanner (Siemens AG, Germany, setting tube voltage 100 kV, tube current 146 mA, slice thickness 1 mm, field of view 477 mm, reconstruction interval 0.6 mm, matrix 512 × 512) for scanning, contrast agent was injected before scanning, and plain and enhanced scans were successively completed; 3-dimensional silhouette image software (Siemens AG) was used to process the scanning data and generate 3-dimensional images of blood vessels.

### 2.7. Assessment indexes

**Figure 4.**
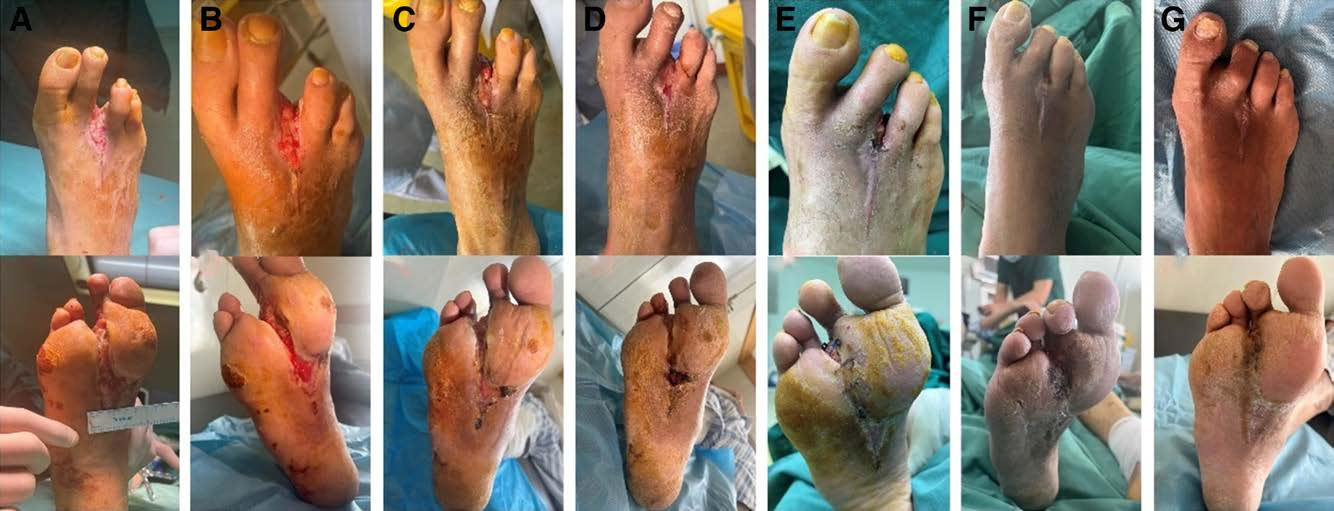
Typical case. a 65-yr-old male patient was admitted due to necrosis of the third toe of the right foot for 2 mo and aggravation with infection of the right foot for 1 wk. He had a history of diabetes for 22 yr and usually had irregular control of blood glucose. Glycosylated hemoglobin was 9.3% and fasting blood glucose was 13.5 mmol/L. Two weeks ago, he had undergone toe amputation of the third toe of the right foot in an outside hospital. In the past 1 wk, he had recurrent ulcers on the dorsum of the right foot and sole. After 2 wk of treatment with blood glucose control, debridement, dressing change, and red light after admission, the foot wound showed no significant reduction; Following the patient’s consent and meticulous preoperative preparation, periosteal distraction of the affected limb and debridement of the right foot were executed. Eventually, after 3 mo of continuous treatment, the right foot wound healed completely. (A) it showed the absence of the third toe of the right foot; (B) it showed the wound condition of the dorsum and sole of the foot on the first day after surgery, and a large amount of fresh granulation tissue was observed on the wound surface; (C) it showed the wound condition of the dorsum and sole of the foot on the seventh day after surgery, and the wound surface began to shrink; (D) it showed the wound condition of the dorsum and sole of the foot on the fourteenth day after surgery, and the wound surface was significantly smaller than before; (E) it showed the wound condition of the dorsum and sole of the foot 1 mo after surgery, and the wound surface of the dorsum of the foot had basically healed, and in order to promote the wound healing of the sole of the foot, we performed compression suture on the wound surface of the sole of the foot to promote the wound growth; (F) it showed the wound healing condition of the right foot 2 mo after surgery, and the wound surface of the dorsum and sole of the foot had basically healed; (G) it showed the wound healing condition of the right foot 3 mo after surgery, and the wound surface of the dorsum of the foot.

The treatment of DFUs is a lengthy process. In this study, all included cases had a follow-up duration exceeding 3 months. Due to the 2-week duration of periosteal distraction, their hospital stays were all over 2 weeks. To ensure complete follow-up, data collection during hospitalization was conducted in the ward. After discharge, patients were instructed to return to the outpatient clinic every 1 to 2 weeks for wound observation and dressing changes, during which necessary follow-up data were collected. Patients were also advised to seek medical attention immediately if there were any changes in their condition. After 3 months of follow-up, all patients underwent routine bilateral lower limb CTA scans to assess neovascularization. To standardize data collection, all case data were gathered by 2 specially trained senior nursing staff members with supervisory roles, with the results averaged between the 2. Finally, data processing and analysis were completed by 2 graduate students from our department. The toe oxygen saturation, ankle-brachial index (ABI), foot skin temperature and visual analogue score (VAS) score of the affected foot before surgery and at 1 day, 7 days, 14 days, 1 month, 2 months, and 3 months after surgery were recorded and compared. Postoperative effect was evaluated using rest root score and CTA 3-dimensional images; the ulcer healing rate, amputation rate and ulcer recurrence rate were also recorded (Fig. 4).

### 2.8. Statistical analysis

SPSS26. statistical software (IBM Corporation, Armonk) was applied to analyze the data, Count data percentage (%), using the chi-square test, and the measurement data conforming to normal distribution were expressed as x ± s. The *t*-test was used for comparison between groups; If normal distribution were violated then date was expressed as median (interquartile spacing), and the Wilcoxon were used for comparison between groups. Categorical data were expressed as the number of patients, the difference between groups was determined by the χ^2^ test, and *P* < .05 was considered statistically significant; Additionally, Cohen *d* was used to represent the effect size, measuring the magnitude of the difference between the 2 groups. Cohen *d* is calculated as the mean difference divided by the standard deviation of the sample. Values of |Cohen *d*| < 0.2 indicate a small effect; 0.2 ≤ |Cohen *d*| ≤ 0.8 indicate a medium effect; and |Cohen *d*| > 0.8 indicate a large effect.^[32]^

## 3. Results

### 3.1. Basic information

A total of 42 patients were included in this study, including 29 males and 13 females, aged (71.17 ± 6.43) and (62–84) years respectively; the average duration of foot ulcers was 3.5 months, and all study subjects underwent conservative medical treatment including blood glucose control, dressing change and oral antibiotics for more than 1 month before admission, which was ineffective and later received surgical treatment in our hospital (Table 1).

**Table 1:** Basic information of patients.

Index				Research object						
				Male		Female				
Gender				29 (69%)		13 (31%)				
Wagner scale										
2				18 (43%)		7 (17%)				
3				8 (19%)		5 (12%)				
4				3 (7)		1 (2%)				
Degree of ischemia										
Ischemic type				23 (55%)		13 (31%)				
Neuro-ischemic type				5 (12%)		1 (2%)				
Neuropathic type				0		0				
Smoking history				20 (48%)		4 (9%)				
Age (yr)				71.17 ± 6.43						
Duration of diabetes (yr)				9 (5–15)						
Ulcer course (mo)				3.5 (2.5–5.0)						
Fasting blood glucose (mmol/L)										
Admission				9.85 (8.72–13.90) 6.79 ± 0.73						
Preoperative										
Hbalc (%)										
Preoperative				8.88 ± 0.98						
Three months after surgery				6.34 ± 0.42						
Hypertension				17 (40%)		8 (19%)				
Vascular diseases of lower				12 (28%)		5 (12%)				
Ulcer site	Toe	18	Sole	14	Dorsum	8	Heel	1	Ankle	1

Abbreviation: Hbalc = glycosylated hemoglobin.

### 3.2. Comparison of oxygen saturation

The oxygen saturation of the affected toe was recorded using a pulse oximeter at various time points, including before the operation, 1 day, 7 days, 14 days, 1 month, 2 months, and 3 months after the operation. The findings indicated a slight increase in oxygen saturation levels in the affected toe 1 day after the operation, but no significant difference was observed between the pre- and postoperative groups (*P* = .83); starting from the seventh day post-operation, a notable increase in oxygen saturation levels was observed in the affected toe in comparison to preoperative levels. Furthermore, statistical analysis revealed significant differences between each subsequent follow-up (*P* < .05); throughout the entire follow-up period, there was a notable increase in oxygen saturation, with a statistically significant difference observed between the latter and previous follow-up (*P* < .05). This suggests that periosteal distraction led to an improvement in blood supply to the affected limb compared to preoperative levels (Table 2).

**Table 2:** Comparison of toe oxygen saturation, ankle-brachial index, foot skin and VAS scores before and after surgery.

Follow-up time	N	oxygen saturation at hallux toe									Ankle-brachial index												Dorsum foot skin temperature												VAS score														
Preoperative	42	79.85 ± 4.10									0.68 (0.60–0.74)												27.15 (26.80–27.50)												5 (5–6)														
1 d after surgery	42	80.04 ± 4.05									0.69 (0.61–0.75)												28.30 (27.90–28.50) ^a^												6 (5–6)*														
Cohen *d*		A									A												A												A														
		–0.04									–0.09												–2.34												–0.95														
7 d after surgery	42	83.19 ± 3.33*,†									0.70 (0.63–0.77)												28.20 (27.90–28.40) ^a^												5 (4–5)†														
Cohen *d*		A	B								A		B										A		B										A			B											
		–0.89	–0.84								–0.38		–0.28										–2.11		0.22										0.48			1.52											
14 d after surgery	42	89.16 ± 1.60*,†,‡									0.71 (0.64–0.78)												29.60 (29.37–30.10)*,†,‡												2 (2–3)*,†,‡														
Cohen *d*		A	B		C						A		B			C							A		B			C							A		B						C						
		–2.98	–2.95		–2.28						–0.45		–0.35			–0.07							–5.16		–3.05			–3.38							3.68		5.13						3.30						
1 mo after surgery	42	90.80 ± 1.72*,†,‡,§									0.72 (0.65–0.79)*,†												30.15 (29.80–30.50)*,†,‡,§												1 (0–1)*,†,‡,§														
Cohen *d*		A	B	C			D				A		B		C				D				A		B		C					D			A	B				C							D		
		–3.47	–3.45	–2.86			–0.98				–0.62		–0.52		–0.25				–0.17				–6.45		–4.31		–4.73					–1.02			6.72	8.80				6.47							3.04		
2 mo after surgery	42	94.38 ± 0.76*,†,‡,§,∥									0.73 (0.67–0.82)*,†,‡,§												31.90 (31.50–32.40)*,†,‡,§,∥												0 (0–1)*,†,‡,§														
Cohen *d*		A	B	C		D		E			A		B		C			D			E		A		B		C			D			E		A				B			C		D				E	
		–4.91	–4.91	–4.62		–4.14		–2.67			–0.86		–0.77		–0.50			–0.43			–0.26		–7.49		–5.90		–6.19			–3.49			–2.85		6.92				9.01			6.69		3.30				0.34	
3 mo after surgery	42	94.80 ± 0.83*,†,‡,§,∥,¶									0.77 (0.72–0.86)*,†,‡,§,∥,¶												32.80 (32.50–33.12)*,†,‡,§,∥,¶												0 (0–1)*,†,‡,§														
Cohen *d*		A	B	C		D			E	F	A	B		C			D			E		F	A	B		C			D		E			F	A		B				C				D	E			F
		–5.04	–5.04	–4.77		–4.40			–2.94	–0.53	–1.39	–1.29		–1.05			–0.98			–0.81		–0.54	–10.72	–9.05		–9.52			–6.11		–5.49			–1.58	7.07		9.20				6.84				3.45	0.48			0.14

Effect size is represented using Cohen *d*: A: effect size compared to preoperative status, B: effect size compared to 1 day post-surgery. C: effect size compared to 7 days post-surgery. D: effect size compared to 14 days post-surgery. E: effect size compared to 1 month post-surgery. F: effect size compared to 2 months post-surgery. Cohen *d* is calculated as the mean difference divided by the standard deviation of the sample, where Cohen *d* < 0.2 indicates a small effect; 0.2 ≤ Cohen *d* ≤ 0.8 indicates a medium effect; and Cohen *d* > 0.8 indicates a large effect.

Abbreviation: VAS = visual analog score.

* Compared with preoperative status, *P* < . 05.

† Compared with 1 d after surgery, *P* < . 05.

‡ Compared with 7 d after operation, *P* < . 05.

§ Compared with 14 d after operation, *P* < . 05.

∥ Compared with 1 mo after operation, *P* < . 05.

¶ Compared with 3 mo after operation, *P* < . 05.

### 3.3. Ankle-brachial index

The ABI was utilized to assess the ratio between the highest systolic blood pressure of the affected foot and ankle and the highest systolic blood pressure of the ipsilateral brachial artery. The normal range for this index is 0.90 to 1.00, with mild ischemia ranging from 0.71 to 0.80, moderate ischemia ranging from 0.50 to 0.70, and severe ischemia being <0.50. A value lower than 0.80 indicates the presence of ischemia in the affected limb^[33]^; In this study, it was observed that the patients exhibited varying degrees of ischemia (0.68, 0.60–0.74) in the affected limb prior to surgery, with moderate ischemia being the predominant manifestation. Following periosteal distraction, there was a general increase in the ABI of the affected limb, with statistically significant changes observed a month post-surgery (*P* = .013) as compared to pre-surgery levels. These findings suggest that periosteal distraction may offer partial improvement in the blood supply to the affected limb; it is worth noticing that at the end of the study, the ABI of the study still showed mild ischemia 0.77 (0.72–0.86), which may be related to the shorter follow-up time (Table 2).

### 3.4. Dorsal foot skin temperature

The temperature of the skin at the midpoint of the dorsum of the foot was measured using a skin thermometer at various intervals, including preoperation, 1 day post-operation, 7 days post-operation, 21 days post-operation, 1month post-operation, 2 months post-operation, and 3 months post-operation. The findings indicate a statistically significant alteration in the skin temperature of the dorsum of the foot on the first day following the operation in comparison to the preoperative state (*P* < .001). However, there was no significant improvement observed on the seventh day post-operation in comparison to the first day (*P* = .18). On the other hand, a significant improvement was observed on the twenty-first day post-operation in comparison to the seventh day (*P* < .001), 1 month after operation was significantly improved compared with 1 month after operation (*P* < .001); 3 months after operation was significantly improved compared with 2 months after operation (*P* < .001); Throughout the follow-up period, the affected limb exhibited an approximate 5°C increase in skin temperature, which was significantly improved by periosteal distraction. These findings suggest that periosteal distraction may enhance blood supply to the foot (Table 2).

### 3.5. Visual analogue score

The VAS score scale was used to record the pain of the affected limb before and after surgery, and the pain score ranged from 0 to 10, which was selected by the patient according to their own pain, from 0 to 10, with 0 indicating no pain, 1 to 3 indicating mild pain, 4 to 6 indicating moderate pain, and 7 to 10 indicating severe pain.^[34]^ The results showed that the preoperative pain of patients in this study was mainly moderate pain; by the end of follow-up, the VAS pain score of patients was significantly lower than before, the postoperative pain of patients was significantly improved, and the difference before and after surgery was statistically significant (*P* < .05), indicating that periosteal distraction could improve the ischemic pain of affected limbs; it is worth noticing that the VAS score of patients at 7 days after surgery was not significantly different from that before surgery (*P* = .057), and the pain relief was not significant, which may be related to the gradual increase of distraction force and the aggravation of pain (Table 2).

### 3.6. Comparison of Michigan neuropathy screening instruments

Michigan neuropathy screening instrument. The appearance of the foot, foot ulcers, ankle reflexes, vibration sensation of the toe, and monofilament touch were assessed in 5 aspects, with a full score of 5 points and a score > 2.5 divided into the presence of peripheral neuropathy^[35]^; this study found that the total score of Michigan score at 3 months after surgery was significantly lower compared with that before surgery, and the difference was statistically significant (*P* < .001), indicating that periosteal distraction could partially improve the peripheral nerve function of patients, but there was no significant difference before and after surgery for the single score of ankle reflexes, vibration sensation of the toe, and monofilament touch (*P* ≥ .05), which indicated that periosteal distraction technique had limited improvement of neurological function in the affected limb (Table 3).

**Table 3:** Comparison of preoperative and 3-mo postoperative Michigan score.

Time	N	Michigan neuropathy screening instrument					
		Appearance of foot	Foot ulcers	Ankle reflex	Vibration sensation at great toe	Monofilament touch	Total
Preoperative	42	1 (0–1)	1 (1–1)	0 (0–1)	0.5 (0–0.5)	0.5 (0–0.5)	3 (1.37–3.62)
3 mo after surgery	42	0 (0–1)	0 (0)	0 (0–1)	0 (0–0.5)	0 (0–0.5)	1 (0.5–2)
*t* (*z*)		–3.67	–7.59	0.00	–0.65	0.00	–4.54
Cohen *d*		0.54	1.11	0.00	0.47	0.27	0.77
*P*		<.001*	<.001*	1.00	.51	1.00	<.001*

Note: Cohen *d* is calculated as the mean difference divided by the standard deviation of the sample, where Cohen *d* < 0.2 indicates a small effect; 0.2 ≤ Cohen *d* ≤ 0.8 indicates a medium effect; and Cohen *d* > 0.8 indicates a large effect.

* indicates statistical significance.

### 3.7. Comparison of lower extremity CTA

The improvement of lower limb blood supply was evaluated by lower limb CTA before operation and 3 months after operation in all study subjects. The results showed that by the end of follow-up, 37 patients showed microcirculation formation in the affected limb by CTA 3 months after operation, lower limb arterioles increased and thickened compared with those before operation, and formed interwoven network, with an improvement rate of 88%; during follow-up, 21 patients had healed foot ulcers 2 months after operation; by the end of follow-up, 38 patients had healed foot ulcers, 5 patients had significantly reduced wound ulcer area, with a wound healing rate of 90%; no amputation or ulcer recurrence occurred during treatment.

## 4. Discussion

DFU refers to lower limb infection, ulcer formation and/or deep tissue destruction in diabetic patients due to combined neuropathy and various degrees of peripheral vascular disease. Because diabetic patients are affected by hyperglycemia for a long time, lower limb vascular sclerosis, vascular wall thickening, decreased elasticity, blood vessels are easy to form thrombi, and aggregate into plaques, resulting in lower limb vascular occlusion, acral nerve injury, resulting in lower limb tissue lesions, which cause ulcers, edema, decay, necrosis, the formation of gangrene.^[36–39]^

At present, there is no uniform conclusion on the treatment of diabetic foot worldwide. In the past, treatment methods commonly included wound dressing changes, debridement, dressings, and vacuum-sealed drainage. However, the treatment cycle was long, which could not effectively improve the blood circulation of the affected limb.^[40–42]^ Interventional therapy is indicated in some elderly patients, but it is not effective in patients with multiple vascular stenoses or occlusions.^[43]^ There is also open surgery for vascular bypass, but the surgical risk is high and long-term may appear luminal restenosis and thrombosis and other problems.^[44]^ At the end of last century, with the popularization of Ilizarov external fixation technique, some researchers found that giving continuous stress stimulation to tissues can lead to neovascularization, so the method of transverse bone transport technique for the treatment of diabetic foot emerged and obtained good results in clinical application^[45–49]^; however, transverse bone transport also has the following drawbacks: The need to cut off the tibia for fenestration increases the patient’s injury and has the problem of nonunion. Large incision, increased incidence of infection complications such as incision infection and osteomyelitis. The process of bone block back pressure was required after operation, which prolonged the wearing time of the fixation; the exposed part of the periosteal distraction device was very large, which increased the psychological burden of the patients and reduced the patient satisfaction. The operation is complex and technically difficult; the operation time is long and aggravates the physical burden of patients; the postoperative patients need to stay in bed for a long time, and the patient compliance is low; therefore, Chinese scholars Zeng Naxin further improved on this basis and proposed a more minimally invasive periosteal distraction technique for the treatment of diabetic foot24. Saulacic et al^[50]^ also proposed the concept of periosteal distraction in 2016. Wang et al^[51]^ reported a case of a chronic diabetic foot patient who was unresponsive to repeated surgical treatments but experienced effective restoration of foot blood supply after using the membrane distraction technique. They suggested that this technique could be employed for chronic ischemic patients with severe below-knee arterial occlusion, thereby providing a “last-mile” blood supply to their feet. Yang et al^[52]^ also compared the efficacy of the membrane distraction technique with tibial transverse bone transfer for treating diabetic foot, finding that the membrane distraction technique had similar effectiveness while offering advantages such as minimal trauma, ease of operation, fewer complications, and convenient nursing care.

In this study, through continuous follow-up of 42 diabetic foot patients treated with periosteal distraction technique, They found that from the first day after surgery, all patients complained of significant fever sensation in the affected limb, and through continuous monitoring of skin temperature, oxygen saturation and other indicators of the affected limb, the skin temperature and oxygen saturation of the affected limb were significantly improved, which ultimately is a manifestation of improved circulation of the affected limb after surgery. At the conclusion of the follow-up period, the majority of the study participants’ wounds had healed, indicating that periosteal distraction can partially enhance circulation in the affected limb, thereby facilitating the resolution of DFUs. However, it is important to acknowledge that diabetic foot is a multifaceted condition that encompasses endocrine, nervous, circulatory, and structural alterations in the foot, and is not solely a vascular disorder. Hence, the utilization of periosteal distraction as a standalone approach for managing diabetic foot is limited in its ability to produce a significant clinical outcome, and serves as a valuable adjunct to surgical interventions. Presently, the clinical management of diabetic foot is predicated on a multifaceted approach that incorporates various modes of comprehensive treatment.

## 5. Limitations

Although periosteal distraction has been successfully implemented as a novel surgical approach in clinical practice, limited research exists regarding its mechanism in treating diabetic foot. Consequently, many domestic and international scholars remain skeptical, highlighting the need for additional fundamental research support. This paper’s study subjects are limited to elderly individuals, potentially introducing selection bias. The sample size included in this study is relatively small. Based on pre-calculated exposure-effect size and statistical power, approximately 278 cases are needed to draw more reliable conclusions. Therefore, future research should increase the sample size to further validate the clinical efficacy of the membrane distraction technique. Additionally, the follow-up period is brief, necessitating further investigation into the long-term efficacy of periosteal distraction for treating diabetic foot. The study solely compares relevant indicators of the affected limb before and after surgery, without conducting a comparative analysis with traditional surgical methods. Furthermore, the results may be influenced by factors such as dressing changes.

## 6. Conclusions

The utilization of periosteal distraction as a novel surgical approach for addressing senile DFUs has demonstrated favorable short-term outcomes and serves as a valuable adjunct to the management of diabetic foot pathology, thereby possessing significant potential for clinical application.

## Author contributions

**Conceptualization:** Sanmao Liu.

**Data curation:** Rui Yang.

**Methodology:** Yue Zhou.

**Validation:** Yue Zhou, Sanmao Liu.

**Visualization:** Sanmao Liu.

**Writing – original draft:** Yunxiang Hu.

**Writing – review & editing:** Yunxiang Hu, Sanmao Liu.
